# CYLD Deubiquitinates RIP1 in the TNFα-Induced Necrosome to Facilitate Kinase Activation and Programmed Necrosis 

**DOI:** 10.1371/journal.pone.0076841

**Published:** 2013-10-02

**Authors:** David M. Moquin, Thomas McQuade, Francis Ka-Ming Chan

**Affiliations:** Department of Pathology, University of Massachusetts Medical School, Worcester, Massachusetts, United States of America; Johns Hopkins School of Medicine, United States of America

## Abstract

**Background:**

Necroptosis/programmed necrosis is initiated by a macro-molecular protein complex termed the necrosome. Receptor interacting protein kinase 1 (RIPK1/RIP1) and RIP3 are key components of the necrosome. TNFα is a prototypic inducer of necrosome activation, and it is widely believed that deubiquitination of RIP1 at the TNFR-1 signaling complex precedes transition of RIP1 into the cytosol where it forms the RIP1-RIP3 necrosome. Cylindromatosis (CYLD) is believed to promote programmed necrosis by facilitating RIP1 deubiquitination at this membrane receptor complex.

**Methodology/Principal Findings:**

We demonstrate that RIP1 is indeed the primary target of CYLD in TNFα-induced programmed necrosis. We observed that CYLD does not regulate RIP1 ubiquitination at the TNF receptor. TNF and zVAD-induced programmed necrosis was highly attenuated in CYLD^-/-^ cells. However, in the presence of cycloheximide or SMAC mimetics, programmed necrosis was only moderately reduced in CYLD^-/-^ cells. Under the latter conditions, RIP1-RIP3 necrosome formation is only delayed, but not abolished in CYLD^-/-^ cells. We further demonstrate that RIP1 within the NP-40 insoluble necrosome is ubiquitinated and that CYLD regulates RIP1 ubiquitination in this compartment. Hence, RIP1 ubiquitination in this late-forming complex is greatly increased in CYLD^-/-^ cells. Increased RIP1 ubiquitination impairs RIP1 and RIP3 phosphorylation, a signature of kinase activation.

**Conclusions/Significance:**

Our results show that CYLD regulates RIP1 ubiquitination in the TNFα-induced necrosome, but not in the TNFR-1 signaling complex. In cells sensitized to programmed necrosis with SMAC mimetics, CYLD is not essential for necrosome assembly. Since SMAC mimetics induces the loss of the E3 ligases cIAP1 and cIAP2, reduced RIP1 ubiquitination could lead to reduced requirement for CYLD to remove ubiquitin chains from RIP1 in the TNFR-1 complex. As increased RIP1 ubiquitination in the necrosome correlates with impaired RIP1 and RIP3 phosphorylation and function, these results suggest that CYLD controls RIP1 kinase activity during necrosome assembly.

## Introduction

Programmed necrosis or necroptosis is a non-apoptotic form of cell death with important functions in pathogen infections, trauma-induced tissue injury, embryonic development and lymphocyte homeostasis [1]. While apoptosis is an immunologically “silent” form of cell death, the release of “danger-associated molecular patterns (DAMPs)” from necrotic cells promotes inflammation [2]. Despite the diametrically opposite effects in physiology, the molecular pathways that regulate apoptosis and programmed necrosis are intimately related. TNF-like death cytokines induce apoptosis by recruiting and activating caspase 8 via the adaptor FADD. The essential programmed necrosis regulators RIP1 and RIP3 are among the substrates of caspase 8 [3,4]. Cleavage of RIP1 and RIP3 inactivates their pro-necrotic kinase activity [5,6]. This inhibitory mechanism is critical to prevent extensive necrosis during embryonic development [7-9], to enforce lymphocyte homeostasis [10,11], and to dampen extensive necrosis-induced inflammation in different tissues [12,13]. Although it is rarely observed, inhibition of programmed necrosis can similarly result in a “switch” to apoptosis in certain cell types [14,15].

The necrosome is a specific and critical cytoplasmic signaling complex for programmed necrosis, since it is not detected when TNF stimulates NF-κB activation or apoptosis [6,16,17]. Besides RIP1 and RIP3, the necrosome also contains the RIP3 substrates mixed lineage kinase domain-like (MLKL) and phosphoglycerate mutase family member 5 (Pgam5) [11,18,19]. Upon TNF stimulation, the essential necrosome component RIP1 is recruited to the TNF receptor 1 (TNFR-1) complex. RIP1 is heavily ubiquitinated in the TNFR-1 signaling complex by the E3 ligases cIAP1 and cIAP2. Polyubiquitinated RIP1 sterically restricts RIP1 from engaging FADD and caspase 8 to inhibit apoptosis [20]. Hence, Smac mimetics or IAP antagonists, which trigger proteasomal degradation of cIAP1 and cIAP2, strongly sensitize cells to TNF-induced apoptosis [21-23]. A similar inhibitory effect of polyubiquitinated RIP1 on necrosome formation has recently been proposed [24].

CYLD is a deubiquitinase and tumor suppressor that is often mutated in tumors affecting the head, neck and skin appendages (reviewed in 25). It was recently identified in a genome-wide screen as a necrosis mediator [26]. RNAi-mediated silencing of CYLD attenuates ROS production and TNF-induced programmed necrosis [14]. Moreover, conditional deletion of FADD in skin epidermis led to extensive necrosis and inflammation that was rescued by crosses to mice carrying a CYLD mutant allele lacking deubiquitinase activity [12]. Although evidence is lacking, CYLD was widely thought to deubiquitinate RIP1 at the membrane-bound TNFR-1 complex to control apoptosis and programmed necrosis [27-30]. However, several pieces of evidence suggest that this model is insufficient to explain the function of CYLD in programmed necrosis. For instance, CYLD is a caspase 8 substrate and cleaved CYLD is rapidly degraded [31]. Since caspase 8 is not recruited to the membrane-anchored TNFR-1 complex where CYLD is supposed to exert its effect [32,33], caspase 8 should not be able to cleave and inhibit CYLD at this complex. Furthermore, in cells treated with Smac mimetics in which RIP1 ubiquitination is inhibited, TNF-induced apoptosis was still protected by siRNA-mediated silencing of CYLD [34]. These results raise the possibility that CYLD may not regulate necrosis by acting as a deubiquitinase within the TNFR-1 signaling complex.

In this study we sought to determine the requirement of CYLD deubiquitinase activity, its relevant target, and the temporal and spatial regulation of the ubiquitination status of this target during TNF-induced necrosis. We reveal that CYLD deubiquitinates RIP1 to facilitate TNF-induced necrosis. In addition, we show that CYLD functions in a distinct signaling compartment to promote programmed necrosis. We show that CYLD does not promote RIP1 deubiquitination within the membrane-associated TNFR-1 complex. Instead, CYLD is recruited to a NP-40 insoluble cytosolic compartment to promote RIP1 deubiquitination within the RIP1-RIP3 necrosome. In the presence of SMAC mimetics or cycloheximide, RIP1-RIP3 binding was delayed, but not abrogated in the absence of CYLD. CYLD deficiency led to hyper-ubiquitinated RIP1 in the necrosome and impaired phosphorylation of RIP1 and RIP3. Hence, CYLD controls the kinetics of RIP1-RIP3 necrosome formation and activation.

## Methods

### Reagents used

Antibodies used in the study were from BD Pharmingen (A20, cIAP1/2, FADD, RIP1, β-actin and TNFR-1), Cell Signaling (p-IκBα and total IκBα), Millipore EMD (K48 and K63-specific ubiquitin antibodies), ProSci (RIP3), Santa Cruz Biotechnology (caspase 8, TRAF2, CYLD) and Invitrogen (CYLD and total ubiquitin antibody). Antibody against human RIP3 has been described before [16]. Necrostatin-1 and zVAD-fmk were obtained from Enzo Life Sciences. CellRox was obtained from Invitrogen. Recombinant human and mouse TNF were obtained from Biosource/Invitrogen. 5-(and 6-)-chloromethyl-2’,7’-dichlorodihydrofluorescein acetyle ester (CM-H _2_DCFDA) and MitoSox were purchased from Invitrogen and used as per manufacturer’s instructions. The Smac mimetic LBW242 was a kind gift of D. Porter (Novartis). Small interference RNAs used in the study are: human A20 (5’-AGUACAAUAGGAAGGCUAAAUAAdTdA-3’, 5’-GCAUGAGUACAAGAAAUGGCAGGAA-3’), human CYLD (5’-CUUAUUUUUAGCAAAGGUUCUACCCUU-3’, 5’-UUGGUUUAUUAUGACUGGAUGAACCUU-3’), mouse CYLD (5’-GGUUUAGAGAUAAUGAUUGGAAAGA-3’, 5’-AGUGUUGAAAGUACAAUUCUCCUGC-3’, 5’-UGAGUAGAUAGCAGUAAAGUCCUCC-3’), human RIP1 (5’-UGCAGUCUCUUCAACUUGAdTdT-3’, 5’-UGCUCUUCAUUAUUCAGUUUGCUCCAC-3’), mouse RIP3 (5’-AAGAUUAACCAUAGCCUUCACCUCCCA-3’, 5’-CCUUCGUUUCCUUUCCUCCUCUCUGUU-3’), human RIP3 (5’-UAACUUGACGCACGACAUCAGGCUGGA-3’, 5’-GCAGUUGUAUAUGUUAACGAGCGGUCG-3’), human TRAF2 (5’-GGACCUGGCGAUGGCUGACdTdT), human TR4 (5’-CCGGAGCUUCCCUCAUUUAdTdT-3’). Mouse RIP1 siRNA sequences have been described [15].

## Tissue Culture

MEFs and L929 cells were grown in DMEM high sucrose medium supplemented with 10% FCS, 2 mM glutamine, 100 units/ml penicillin, and 100 µg/ml streptomycin. HT-29 and Jurkat cells were grown in McCoy’s 5A medium and RPMI1640 media, respectively, supplemented with 10% FCS, 2 mM glutamine, 100 units/ml penicillin, and 100 µg/ml streptomycin. Stable clones of HT-29 cells were generated by transfection using lipofectamine 2000 (Invitrogen) of CYLD-specific and scrambled shRNAs from Thermoscientific/Open Biosystems. Clones were selected by puromycin selection and checked for GFP expression via flow cytometry. GFP^+^ clones were tested for reduction of CYLD protein expression and used for subsequent experiments.

### Cell death assays

Wild type and CYLD^-/-^ MEFs (kind gift from S.C. Sun [35]) were treated with 0.5-1 µg/ml cycloheximide (CHX) and 20 µM zVAD-fmk where indicated for 1 hour prior to stimulation with the indicated amounts of recombinant mouse TNFα (rmTNF, TRAIL, FasL, or staurosporine. In some instances, 5-10 µM the Smac mimetic LBW242 was used. For FADD and caspase-8 deficient TNFR2^+^ Jurkat Cells [5], necrosis was induced by addition of the indicated amounts of recombinant human TNF (rhTNF). HT-29 cells were treated with 5-10 µM LBW242 and 20 µM zVAD-fmk for 1 hour prior to stimulation with 10-100 ng/ml rhTNF. Cell death was determined by flow cytometry with propidium iodide staining or by CellTiter 96® AQueous One Solution Cell Proliferation Assay (Promega).

### Transfection of DNA Plasmids and siRNA

Jurkat cells were transfected with 150 nM of the indicated siRNA. For L929 cells, 20 nM siRNA was used per transfection. All siRNA transfections were performed using the HiPerfect transfection reagent (Qiagen) as per manufacturer’s protocols. Forty-eight hours later, cells were stimulated with TNF to induce necrosis.

For DNA plasmid transfection into MEFs, 2.5 x 10^5^ cells per well of 12-well plate were plated the day before transfection. Transfection was performed using the Lipofectamine LTX transfection reagent (Invitrogen) as per manufacturer’s protocol. CYLD deletion mutants were generated by PCR amplification and cloning into pEGFP-C1 vector. Integrity of the mutant clones was confirmed by sequencing.

For 293T cells, three hours prior to transfection cells were plated at 3 x 10^5^ cells/well of 12-well plate. Cells were subsequently transfected with 1 μg/well (12-well plate) of the indicated plasmid DNA using the Fugene6 or Fugene HD transfection reagent (Roche) as per manufacturer’s protocol.

### Immmunoprecipitations and Western Blots

For immunoprecipitations in MEFs, six million cells on two 10 cm tissue culture dishes were used per sample. For HT-29 cells, one plate of cells was used per sample. For Jurkat cells, 100-150 million cells were used. Cells were harvested and lysed in either Complex II lysis buffer (150 mM NaCl, 20 mM Tris-Cl [pH 7.5], 1% NP-40, 1 mM EDTA, 3 mM NaF, 1 mM β-glycerophosphate, 1 mM sodium orthovanadate) or RIPA lysis buffer (150 mM sodium chloride, 1% NP-40, 0.5% sodium deoxycholate, 0.1% SDS, and 50 mM Tris pH 8.0) supplemented with 5 µM iodoacetamide, 2 µM *N*-ethylmaleimide, 1X *Complete* protease inhibitors (Roche) and Phosphatase inhibitor cocktail I (Sigma). For denaturing IPs, cells were lysed in 1% SDS, 50 mM TrisCl pH7.4, 5 mM EDTA, 10 mM DTT, 15 U/ml DNase I supplemented with Complete protease inhibitors (Roche), and phosphatase inhibitor cocktail II (Sigma). After clearance with Sepharose 6B beads, lysates were mixed with specific immunoprecipitation antibodies as indicated for 4 hours to overnight at 4°C. Immune complexes were then washed, boiled, and resolved on a 4-12% Bis-Tris NuPAGE gel (Invitrogen). For sequential IP, the washed immune complex was resuspended in RIPA buffer supplemented with 6 M urea. After rotation at room temperature for 30 minutes, lysates were diluted 10-fold in RIPA buffer without urea and incubated with different ubiquitin antibodies.

For differential centrifugation, cell lysates were centrifuged at 1,000g for 10 minutes. The resulting supernatants were centrifuged at 10,000g for 10 minutes. The resulting pellet was washed in 1 ml lysis buffer and centrifuged at 10,000g for an additional 10 minutes yielding the P10 fraction. The supernatant following first spin at 10,000g was transferred to a new tube and spun at 25,000g. The resulting supernatant and pellets were the S25 and P25 fractions.

### In vitro kinase assays

The IP complexes were incubated for 30 minutes at 30°C in kinase reaction buffer (20 mM HEPES [pH 7.5], 2 mM DTT, 1 mM NaF, 1 mM Na _3_VO_4_, 20 mM β-glycerophosphate, 20 mM MgCl_2_, 20 mM MnCl_2_, 1 mM EDTA, and 200-400 µM ATP) supplemented with 10 µCi [^32^P] γ-ATP and 5 µM Histone H1. Reactions were quenched by boiling in sample loading buffer. Phosphorylation of Histone H1 was visualized by autoradiography.

**Figure 1:**
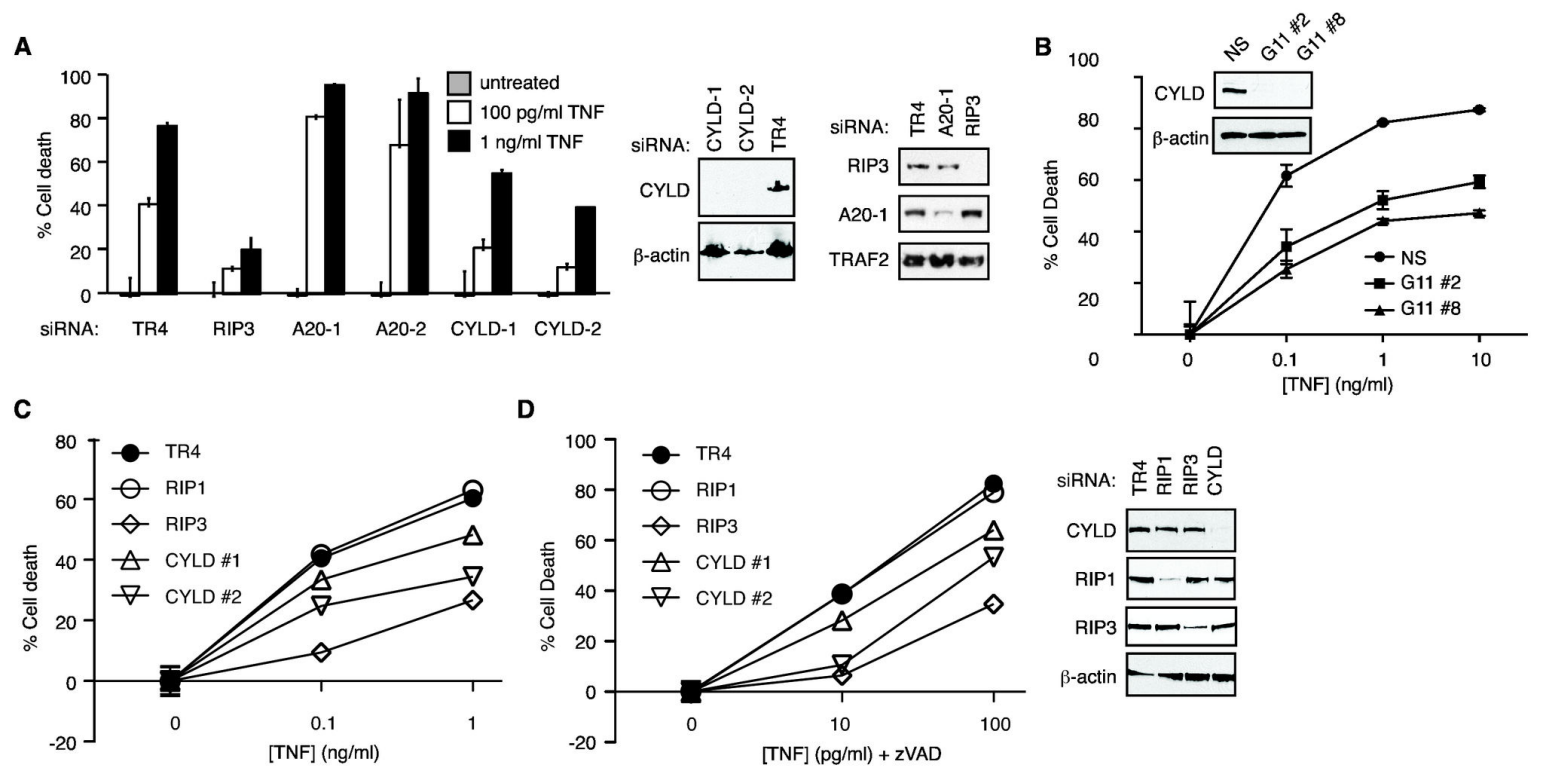
Partial protection against TNF-induced necrosis by CYLD siRNAs. (**A**) FADD-deficient Jurkat cells were transiently transfected with the indicated siRNAs. Forty-eight hours post-transfection, the cells were treated with TNF to induce necrosis. The percentage cell death was determined by propidium iodide staining and flow cytometry. Western Blots on the right show the efficiency of gene silencing. Representative of three experiments. (**B**) HT-29 cells were stably transfected with either non-specific shRNA (control) or CYLD targeting shRNA (clones: G11-2 and G11-8). Cells were treated with TNF, LBW242 and zVAD-fmk. Cell viability was determined by MTS assay (Promega). Western blot shows that expression of CYLD was inhibited in the two selected clones, G11 #2 and G11 #8. Representative of three experiments. (**C**-**D**) L929 cells were transfected with the indicated siRNAs. Necrosis was induced with (**C**) TNF or (**D**) TNF and zVAD-fmk. Cell death was determined by MTS assay. The panel on the right shows the reduction in protein expression of the siRNA transfected cells. Representative of two experiments.

## Results

### CYLD promotes but is not essential for TNF-induced programmed necrosis

Assembly of the RIP1-RIP3 necrosome is a key event in programmed necrosis. The current model predicts that RIP1 in the TNFR-1 complex has to be deubiquitinated before it can engage RIP3 to form the cytosolic necrosome [36]. Polyubiquitinated RIP1 is a known substrate of CYLD [37], a deubiquitinase that has been shown to participate in necrosis signaling [26]. In agreement with previous results, silencing of CYLD expression by siRNAs protected FADD-deficient Jurkat cells from TNF-induced programmed necrosis compared with cells transfected with the control siRNA against TRAIL-R4 (TR4) (Figure 1A). In contrast, siRNA silencing of A20, another RIP1 deubiquitinase [38,39], enhanced rather than inhibited TNF-induced programmed necrosis (Figure 1A). Surprisingly, we consistently observed weaker protection by siRNA against CYLD compared with those against RIP1 or RIP3 (Figure 1A). This is in contrast to the expectation that CYLD is an essential regulator of necrosis upstream of RIP1 and RIP3 activation [29,40]. Similarly, HT-29 cells with stable knock-down (kd) of CYLD also exhibited residual necrosis induced by TNF, zVAD and Smac mimetics (SM) (Figure 1B).

**Figure 2:**
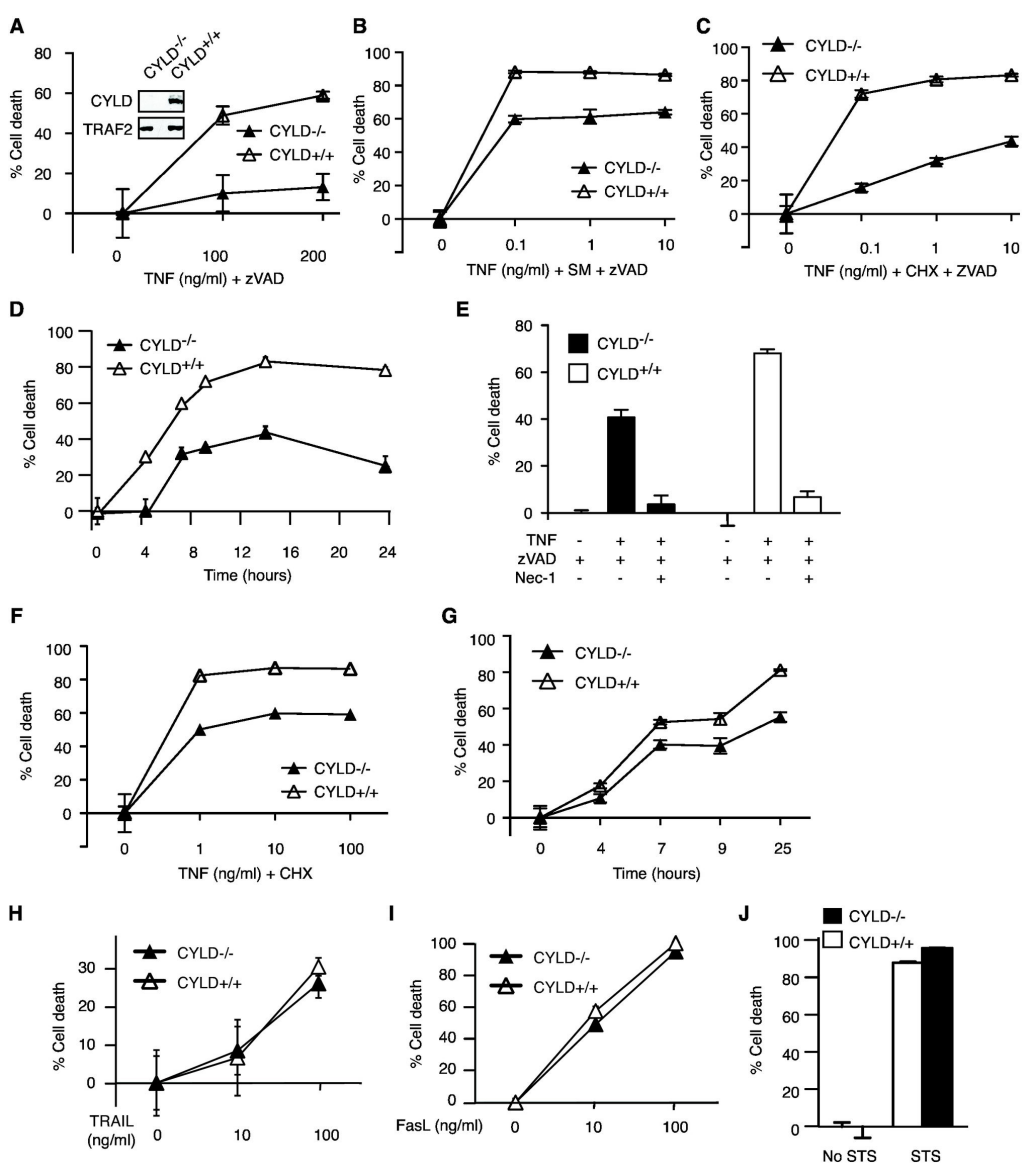
RIP1-dependent necrosis occurs in CYLD^-/-^ MEFs. Wild type (CYLD^+/+^) and CYLD^-/-^ MEFs were treated with (**A**) TNF and zVAD, (**B**) TNF, Smac mimetic and zVAD, or (**C**) TNF, cycloheximide (CHX) and zVAD-fmk for 12 hours. Cell death was determined by staining with propidium iodide (PI) and analyzed via flow cytometry. The inset shows loss of CYLD expression in CYLD^-/-^ MEFs using a C-terminal specific CYLD antibody. In (**D**), cells were treated with 10 ng/ml TNF, CHX and zVAD-fmk and cell death was measured at the indicated times following treatment. Representative of three experiments. (**E**) Nec-1 inhibited TNF-induced necrosis in CYLD^-/-^ MEFs. Representative of two experiments. (**F**) CYLD^+/+^ and CYLD^-/-^ MEFs were treated with TNF and CHX for 12 hours. Cell death was determined by PI staining and flow cytometry. Representative of three experiments. (**G**) MEFs were treated with 10 ng/ml TNF and CHX for the indicated times. Representative of two experiments. (**H**-**J**) MEFs were treated with (**H**) TRAIL, (**I**) FasL or (**J**) staurosporine (STS) for 12 hours. Cell death was determined by PI exclusion and flow cytometry.

L929 cells are widely used to study necrosis because they undergo necrosis exclusively in response to TNF. Similar to Jurkat and HT-29 cells, CYLD siRNAs provided partial protection compared with RIP3 siRNA against TNF or TNF and zVAD induced necrosis in L929 cells (Figure 1C-D). Consistent with previous reports [15,41], siRNA-mediated silencing of RIP1 did not protect L929 against TNF-induced or TNF and zVAD-induced necrosis (Figure 1C-D). The molecular basis for this RIP1-independent necrosis in L929 cells is unknown at present. However, similar RIP1-independent necrosis has recently been observed in cells with over-expression of RIP3 [42]. Thus, high RIP3 expression level could circumvent the requirement for RIP1 in necrosis signaling.

**Figure 3:**
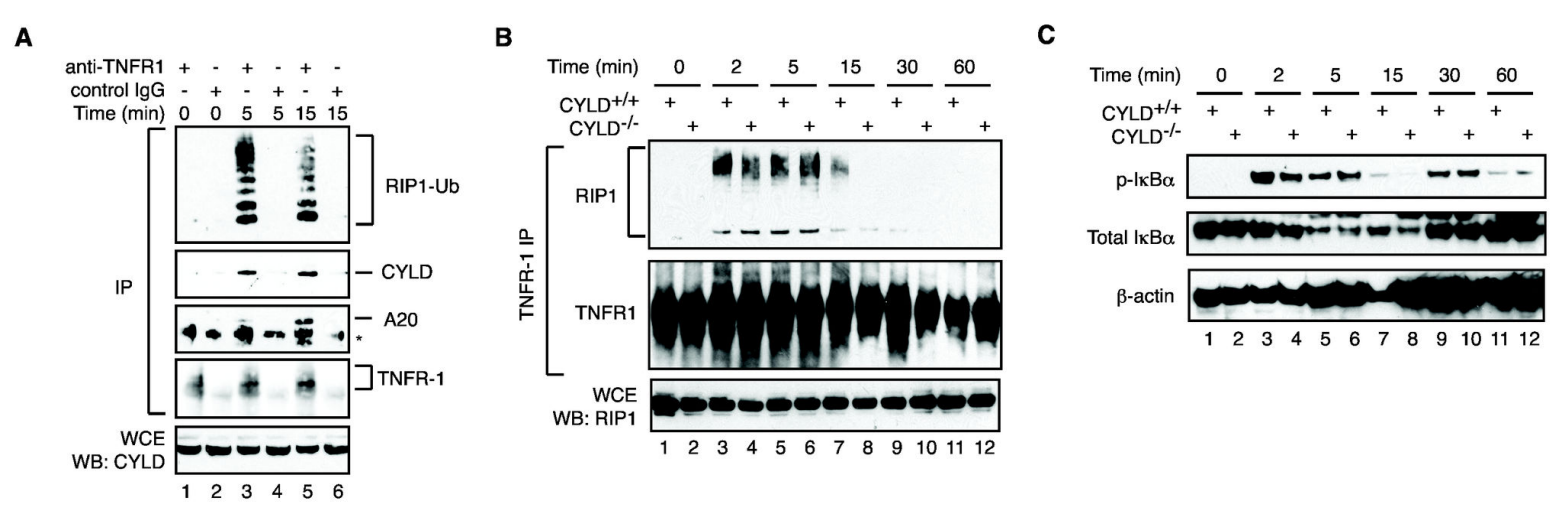
CYLD does not regulate RIP1 ubiquitination within the TNFR1 complex. (**A**) Recruitment of CYLD and A20 to the TNFR-1 complex. TNFR1 complex was purified from TNF, zVAD and CHX treated cells by immunoprecipitation (IP). The recruitment of RIP1, CYLD and A20 was assessed via Western Blot. Control IPs with isotype-matched IgG were included to show the specificity of binding to TNFR-1. (**B**) CYLD^+/+^ and CYLD^-/-^ MEFs were treated with TNF for the indicated times. Recruitment of polyubiquitinated RIP1 to TNFR-1 was determined by Western Blot. (**C**) IκBα phosphorylation and degradation was normal in CYLD^-/-^ MEFs.

The residual cell death observed in CYLD knock-down cells was not due to insufficient inhibition of CYLD expression by the siRNAs, since CYLD^-/-^ mouse embryonic fibroblasts (MEFs) also exhibited reduced but residual necrosis in response to TNF and zVAD (Figure 2A), TNF, SM and zVAD (Figure 2B), or TNF, cycloheximide (CHX) and zVAD (Figure 2C). While necrosis was greatly reduced in TNF and zVAD treated CYLD^-/-^ MEFs, the inclusion of CHX or SMAC mimetics led to necrosis of CYLD^-/-^ MEFs at levels closer to that of wild type cells. A comparison of the kinetics of necrosis in WT MEFs and CYLD^-/-^ MEFs revealed that there was a delay in the onset of necrosis in the absence of CYLD, followed by residual necrosis that is attenuated in magnitude (Figure 2D). The residual necrosis in CYLD^-/-^ MEFs was completely inhibited by the RIP1 kinase inhibitor necrostatin-1 (Figure 2E), indicating that CYLD is not essential for TNF- and RIP1-dependent necrosis. CYLD^-/-^ MEFs also exhibited moderate resistance to apoptosis induced by TNF and CHX (Figure 2F-G). By contrast, CYLD^-/-^ cells responded normally to apoptosis induced by TRAIL, FasL or staurosporine (Figure 2H-J). Hence, while CYLD has a significant role in TNF and zVAD induced necrosis, it plays only an auxiliary role to promote TNF and zVAD-induced necrosis in the presence of SM or CHX.

### CYLD does not control RIP1 ubiquitination within the TNFR-1 complex

These results led us to re-evaluate the role of CYLD in programmed necrosis. RIP1 recruited to TNFR-1 was heavily ubiquitinated (Figure 3A). Although CYLD was recruited to TNFR-1 in a ligand-dependent manner (Figure 3A, compare lanes 1, 3 and 5), RIP1 ubiquitination was not affected in CYLD^-/-^ MEFs (Figure 3B, compare lanes 3-6). Polyubiquitinated RIP1 within the TNFR-1 signaling complex is important for assembly and activation of the IKK complex. Consistent with the normal RIP1 ubiquitination status, IκBα phosphorylation and degradation was normal in CYLD^-/-^ MEFs (Figure 3C). The normal TNFR-1 associated RIP1 ubiquitination in CYLD^-/-^ cells was not due to compensatory effect of another RIP1 deubiquitinase A20 that was recruited to the TNFR-1 complex (Figure 3A, lane 5), since A20 siRNA enhanced rather than decreased TNF-induced necrosis (Figure 1A).

**Figure 4:**
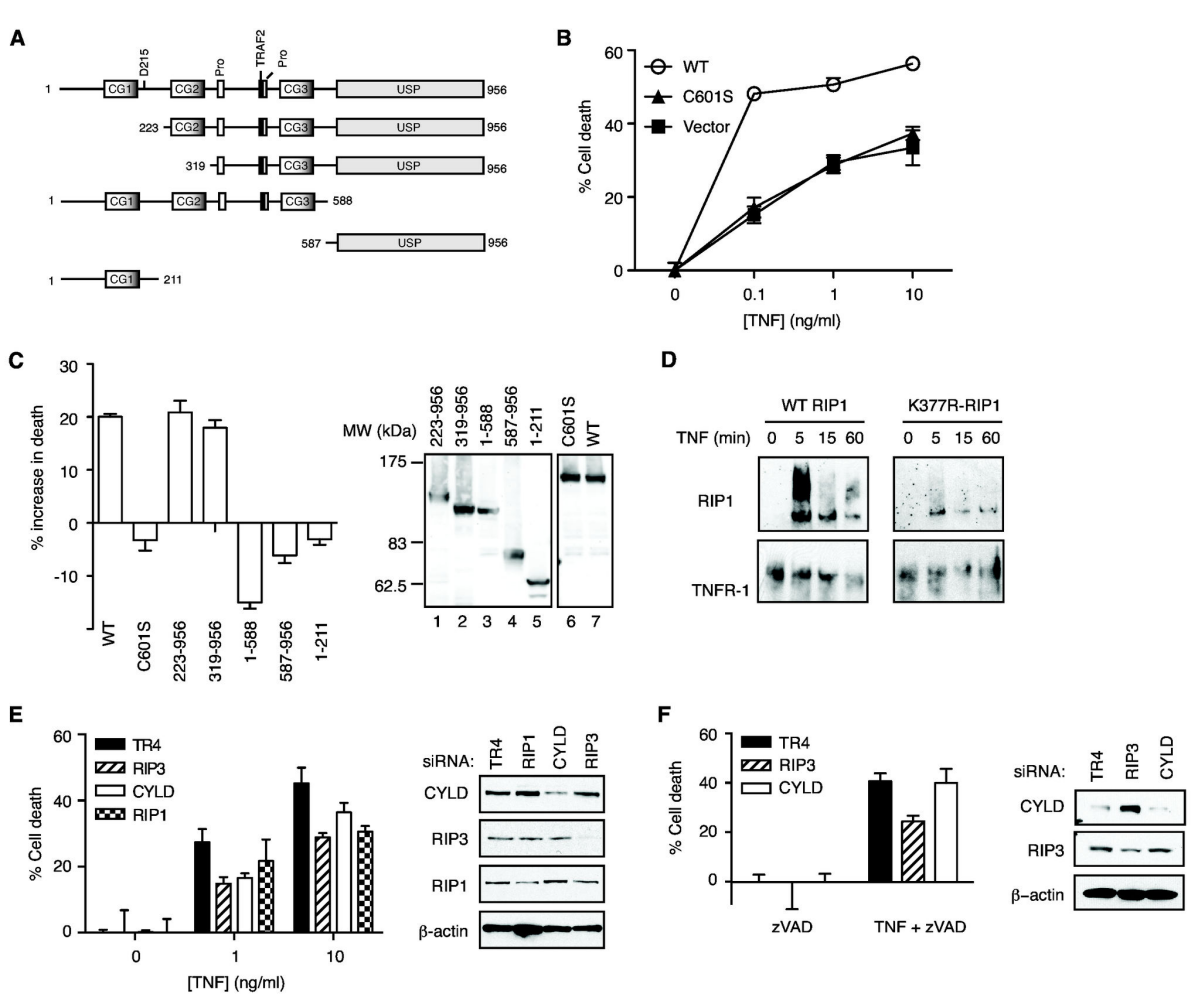
Poly-ubiquitinated RIP1 is the major substrate for CYLD in TNF-induced necrosis. (**A**) Schematic diagram of wild-type CYLD and deletion mutants used in the experiments. (**B**-**C**) CYLD^-/-^ MEFs were transiently transfected with the indicated GFP-tagged CYLD. Necrosis was induced with TNF, CHX and zVAD-fmk. Cell death was determined in the GFP^+^ population by PI staining and flow cytometry. The panel on the right of (**C**) shows that the GFP-tagged CYLD mutants were of the correct sizes. (**D**) RIP1-deficient Jurkat cells reconstituted with wild type RIP1 or RIP1-K377R were stimulated with TNF. The recruitment of RIP1 to TNFR-1 was determined by Western blot. (**E**) RIP1-deficient Jurkat cells stably expressing either WT RIP1-GFP or (**F**) RIP1-K377R-GFP mutant were transfected with the indicated siRNAs. Necrosis was induced with TNF and zVAD-fmk. Cell death was determined as described before [48]. The Western blots provide validation of siRNA knock-down efficiency.

### Deubiquitination of RIP1 by CYLD facilitates programmed necrosis

Certain deubiquitinases, such as A20, have been shown to regulate cell signaling independent of their enzymatic activity [39]. Because RIP1 ubiquitination in the TNFR-1 complex was normal in CYLD^-/-^ cells, we asked if CYLD similarly regulates programmed necrosis independent of its deubiquitinase activity. We found that expression of GFP-tagged wild type CYLD, but not the deubiquitinase inactive mutant C601S, enhanced TNF-induced programmed necrosis in CYLD^-/-^ MEFs (Fig. 4A-C). Truncation mutants lacking the carboxyl terminal ubiquitin specific protease (USP) domain also failed to restore programmed necrosis in CYLD^-/-^ MEFs (Figure 4C). By contrast, the first and second CAP-Glycine (CG1 and CG2) domains were dispensable for programmed necrosis (Figure 4C). The negative values in percentage change in cell death in certain mutants were due to proliferation of the transfected populations. Hence, the deubiquitinase function of CYLD is required to promote TNF-induced programmed necrosis.

Since the deubiquitinase function of CYLD is required for programmed necrosis and yet RIP1 ubiquitination within the TNFR-1 complex was normal in CYLD^-/-^ cells, we asked if RIP1 is indeed the major substrate of CYLD during programmed necrosis. Expression of the RIP1 mutant K377R inhibited TNF-dependent RIP1 poly-ubiquitination in the TNFR-1 complex (Figure 4D) [43,44]. RIP1-deficient Jurkat cells reconstituted with wild type RIP1 were protected from TNF-induced necrosis by siRNAs against RIP1, RIP3 and CYLD (Figure 4E). Strikingly, unlike cells expressing wild type RIP1, cells that express the K377R mutant were not protected from TNF-induced necrosis by CYLD siRNA (Figure 4E compare left and right graphs). In contrast, RIP3 siRNA reduced programmed necrosis in these cells (Figure 4E). Hence, we conclude that RIP1 is indeed the major substrate of CYLD in programmed necrosis.

**Figure 5:**
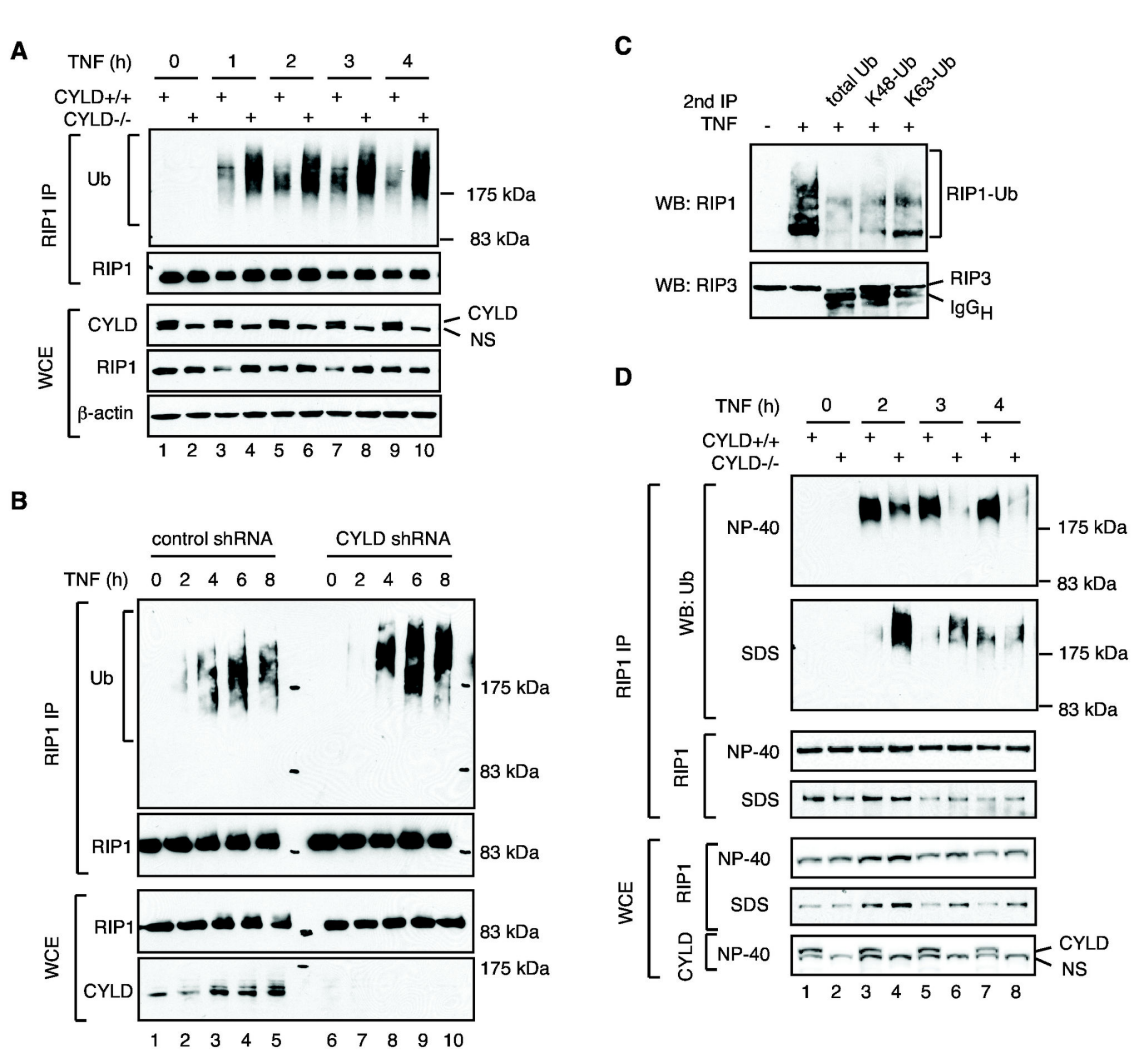
CYLD regulates RIP1 ubiquitination in the necrosome. (**A**-**B**) Increased RIP1 ubiquitination in the absence of CYLD. (**A**) CYLD^+/+^ and CYLD^-/-^ MEFs or (**B**) control and CYLD knock-down HT29 cells were treated with TNF, the Smac mimetic LBW242 [35] and zVAD-fmk for the indicated times. Total lysis in 1% SDS was performed as described in the methods. The level of RIP1 ubiquitination was examined by Western Blot. NS: non-specific band. (**C**) Necrosome-associated RIP1 contains ubiquitin chains of different linkage types. Cells were treated with TNF and zVAD-fmk for 3 hours or left untreated. RIP3 immune complexes were denatured in urea, followed by immunoprecipitation with the indicated antibodies against ubiquitin. (**D**) Selective accumulation of poly-ubiquitinated proteins in RIP1 complexes in the NP-40 insoluble compartment. WT MEFs or CYLD^-/-^ MEFs were treated to undergo necrosis with TNF, zVAD-fmk, and CHX for the indicated times. Cells were lysed in NP-40 lysis buffer and insoluble material was solubilized with SDS. RIP1 was immunoprecipitated from both fractions followed by Western blot with the indicated antibodies.

### CYLD regulates RIP1 ubiquitination in a spatially and temporally distinct compartment

Since RIP1 is the major substrate of CYLD and yet RIP1 ubiquitination within the TNFR-1 signaling complex was not affected in CYLD^-/-^ cells, we assessed the possibility that CYLD might regulate RIP1 ubiquitination in a different signaling compartment. We prepared cell lysates by boiling in 1% SDS. This method allows us to remove most of the RIP1 binding proteins prior to RIP1 immunoprecipitation. Using this method, we detected RIP1 polyubiquitination in a TNF-dependent manner in both wild type and CYLD^-/-^ cells (Figure 5A). These modifications were detected several hours after TNF stimulation when RIP1 has already been dissociated from the TNFR-1 signaling complex (Figure 3B). In contrast to RIP1 recruited to the TNFR-1 signaling complex, RIP1 isolated from CYLD^-/-^ cells using this method exhibited higher levels of ubiquitination than wild type cells at all time points examined (Figure 5A). Moreover, the length of the polyubiquitinated species was longer in the CYLD^-/-^ cells compared with that in wild type cells. Similar results were obtained in HT29 cells with stable expression of shRNA against CYLD (Figure 5B). Hence, RIP1 was indeed hyper-ubiquitinated in CYLD^-/-^ cells after dissolution of the TNFR-1 complex.

Ubiquitin-like modifications of RIP1 and RIP3 were previously detected in the late-forming cytosolic necrosome [16]. This suggests that CYLD might regulate RIP1 ubiquitination within the necrosome. To ascertain this possibility, we performed sequential immunoprecipitation, first with RIP3, followed by denaturation in urea and immunoprecipitation with different ubiquitin antibodies. Consistent with our previous observations [45], the RIP1-RIP3 necrosome was stable in urea. Although the different ubiquitin antibodies exhibited differential efficiency in immunoprecipitation, we found that RIP1 within the necrosome was indeed modified by ubiquitination via K48 and K63 linkages (Figure 5C). To determine the role of CYLD in necrosome ubiquitination, we performed differential detergent lysis, first with NP-40, followed by extraction of the NP-40 insoluble materials with SDS. RIP1 ubiquitination was reduced in CYLD^-/-^ cells compared with wild-type cells in the NP-40 fractions (Figure 5D, compare lanes 3 and 4, 5 and 6, and 7 and 8). In contrast, in the NP-40 insoluble, SDS-soluble fraction, RIP1 was hyper-ubiquitinated in CYLD^-/-^ cells (Figure 5D, compare lanes 3-4 and 5-6). Because the necrosome preferentially accumulates in NP-40 insoluble compartment [45], these results suggest that CYLD regulates RIP1 ubiquitination after necrosome assembly.

### CYLD controls the kinetics of necrosome formation and activation

**Figure 6:**
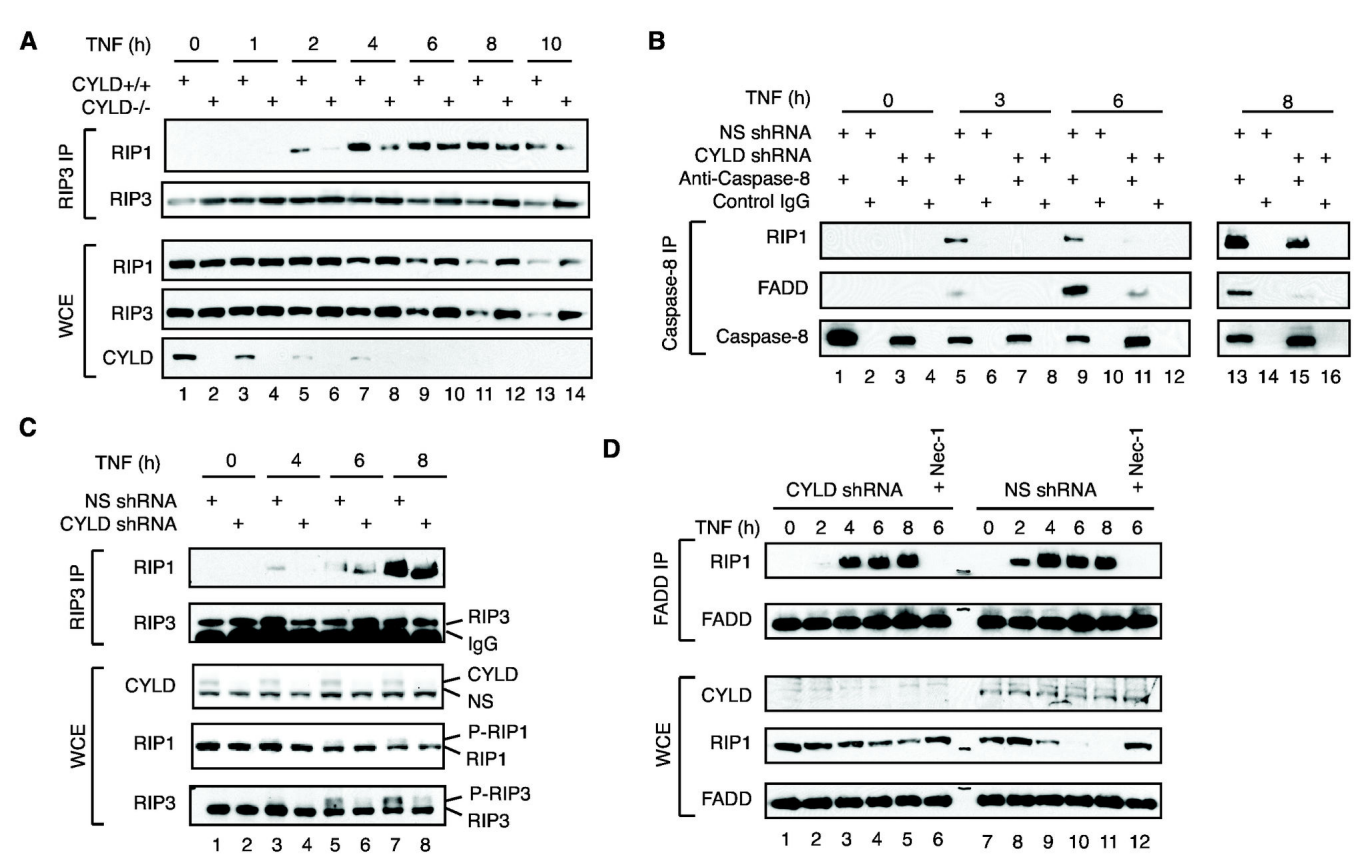
CYLD regulates the kinetics of RIP1-RIP3 necrosome assembly. (**A**) WT or CYLD^-/-^ MEFs were treated with TNF, LBW242 and zVAD-fmk for the indicated times. Recruitment of RIP1 to RIP3 was determined by Western Blot. (**B**) HT-29 cells expressing non-specific shRNA (NS) or CYLD shRNA were treated with TNF, LBW242 and zVAD-fmk. RIP3 complexes were immunoprecipitated and RIP1 association was determined by Western Blot. (**C**) HT-29 cells were treated with TNF, the SM LBW242 and zVAD-fmk. Caspase-8 complexes were immunoprecipitated, and recruitment of RIP1 and FADD was determined by Western blot. (D) HT-29 cells were treated with TNF, Smac mimetic and zVAD-fmk. FADD complexes were immunoprecipitated, and recruitment of RIP1 was determined by Western blot.

We next examined the consequence of this hyper-ubiquitinated form of RIP1 in necrosis signaling. Surprisingly, we found that the assembly of the RIP1-RIP3 necrosome was delayed, but not abolished in CYLD^-/-^ MEFs (Figure 6A, lanes 5-8). Similar observations were made in CYLD knock-down HT-29 cells (Figure 6B, compare lanes 3 and 4, 5 and 6, and 7 and 8). Consistent with the requirement of CYLD for TNF-induced apoptosis (Figure 2), recruitment of RIP1 and FADD to caspase-8 was similarly delayed in CYLD knock-down HT29 cells (Figure 6C, compare lanes 5 and 7, 9 and 11, and 13 and 15). Similarly, association between RIP1 and FADD was delayed in CYLD knock-down HT-29 cells (Figure 6D, compare lanes 2 and 8), but caught up to control cells at later time-points (Figure 6D, compare lanes 3-5 with lanes 9-11). Nec-1 blocked the association between RIP1 and FADD in both cell-types demonstrating that RIP1 kinase activity is required for complex formation as previously reported (Figure 6D, compare lanes 6 and 12). Thus, CYLD promotes, but is not essential for assembly of the RIP1-RIP3 necrosome.

**Figure 7:**
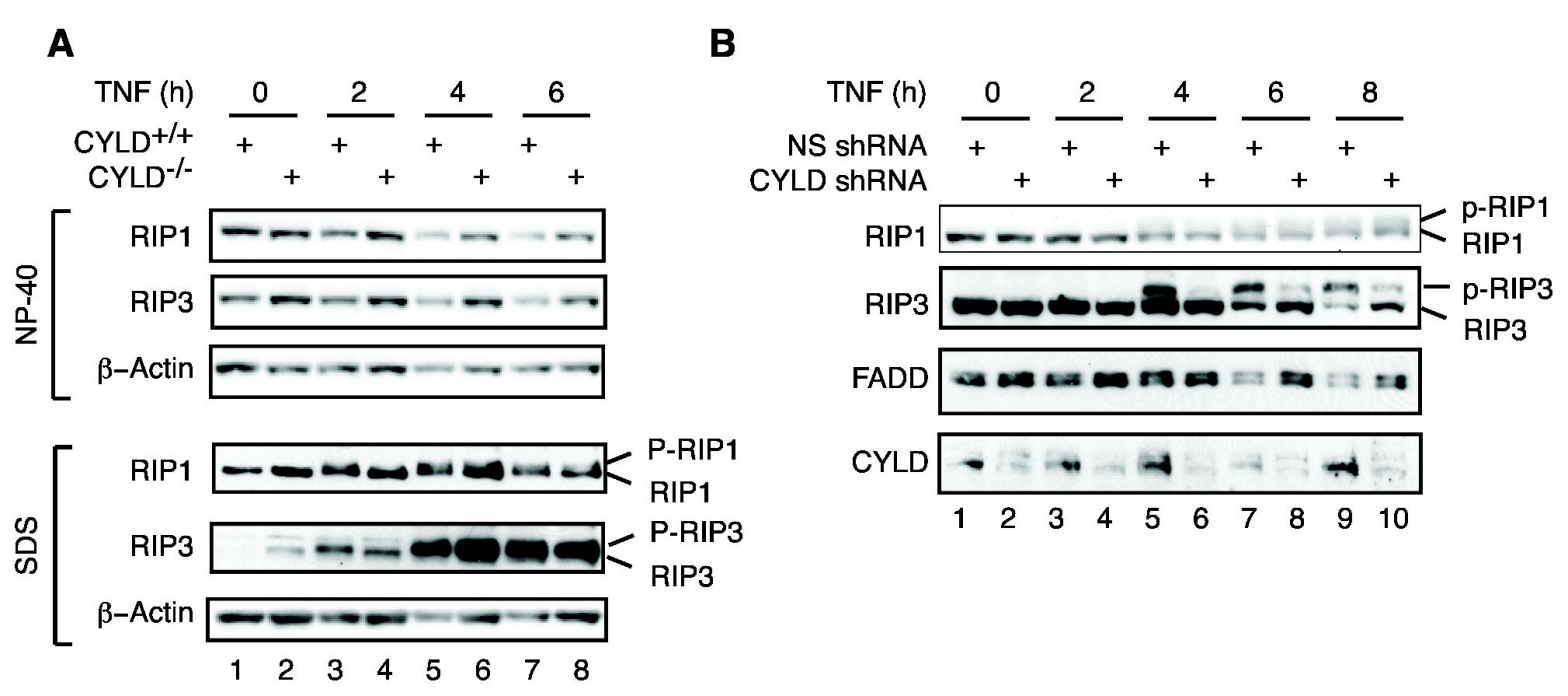
Impaired RIP1 and RIP3 phosphorylation in the absence of CYLD. (**A**) MEFs were treated with TNF, zVAD-fmk and the LBW242. Cell lysates were extracted by sequential detergent lysis in NP-40 and SDS as described in methods. RIP1 and RIP3 in each fraction were examined by Western blot. Note that phosphorylated RIP1 and RIP3 (p-RIP1 and p-RIP3) were exclusively detected in the SDS fractions. (**B**) HT-29 cells stably expressing non-specific (NS) shRNA or CYLD shRNA were treated with TNF, LBW242 and zVAD-fmk for the indicated times. Unmodified and phospho-RIP1 and phospho-RIP3 were analyzed by Western Blot.

Sequential detergent extraction with NP-40 and SDS confirmed that RIP1 and especially RIP3 accumulated in the NP-40 insoluble, SDS fraction in a TNF-dependent manner (Figure 7A, bottom panels). Active, phosphorylated RIP1 and RIP3 as indicated by mobility shift were detected in the NP-40 insoluble SDS fraction as early as 2 hours post stimulation in wild type cells (Figure 7A, lanes 3). By contrast, appearance of phospho-RIP1 and phospho-RIP3 was not apparent until 4 hours post-stimulation in CYLD^-/-^ cells (Figure 7A, lane 6). The delayed phosphorylation of RIP1 and especially RIP3 was even more apparent in CYLD knock-down HT29 cells (Figure 7B, lanes 5-10).

**Figure 8:**
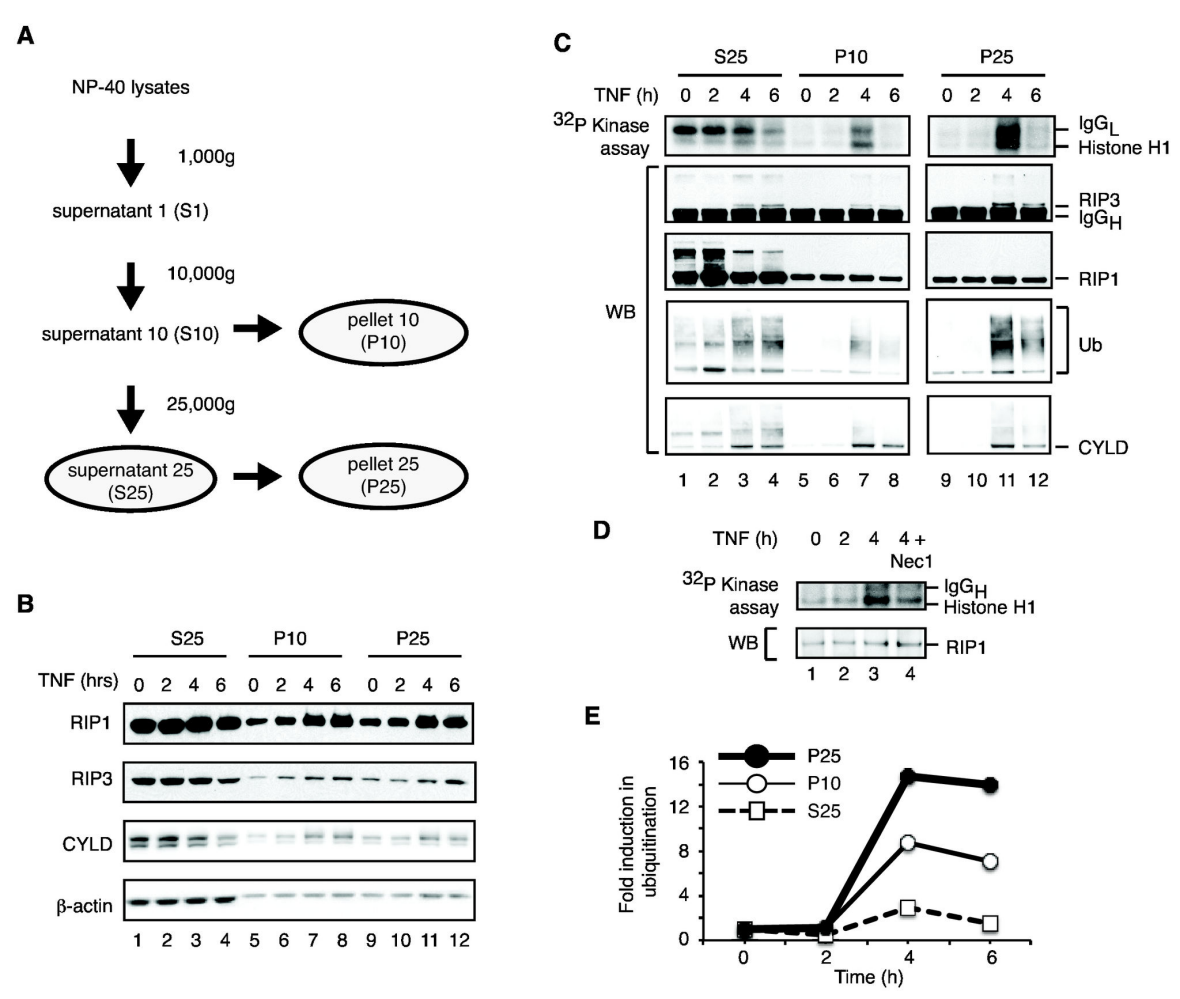
Induction of RIP1 associated kinase activity in the NP-40 insoluble compartment. (**A**) Schematic flowchart showing the procedures by which the different NP-40 soluble and insoluble fractions were obtained. (**B**) Accumulation of necrosis signaling adaptors in the NP-40 insoluble fractions. The indicated fractions were analyzed for levels of RIP1, RIP3 and CYLD in response to necrosis induction by TNF. β-actin was used as internal control. (**C**) The kinase activity of the necrosome is selectively activated in the NP-40 insoluble fractions. Cell lysates were subjected to differential centrifugation as described in (**A**). The RIP1 immune complexes were subjected to *in* *vitro* kinase assays using histone H1 as substrate. RIP1 ubiquitination and recruitment of RIP3 and CYLD were determined by Western blot (lower panels). (**D**) Induction of kinase activity in the NP-40 insoluble fraction is partially dependent on RIP1 kinase activity. The P10 fractions were prepared and RIP1 immune complexes were isolated. Where indicated, 30 µM of Nec-1 was added to the *in* *vitro* kinase assay. (**E**) Densitometry quantification of RIP1 ubiquitination in the different fractions in (**C**).

The preferential phosphorylation in the SDS fraction suggests that the kinase activity of the necrosome was activated in this compartment. Because SDS inhibited RIP1 and RIP3 kinase activity in vitro (data not shown), we used differential centrifugation to separate the NP-40 insoluble fraction to determine its kinase activity (Figure 8A). We confirmed that similar to the differential detergent lysis, the insoluble pellet fractions obtained with this method exhibited ligand-dependent accumulation of the necrosome components RIP1, RIP3 and CYLD (Figure 8B, compare lanes 5-8 and 9-12). Although TNF-induced RIP1-RIP3 necrosome was detected in both NP-40 soluble and insoluble fractions (Figure 8C, second panel, lanes 3-4, 7-8 and 11-12), TNF-induced and RIP1-associated kinase activity was detected only in the NP-40 insoluble fractions (Figure 8C, top panel, lanes 5-8 and 9-12). By contrast, although the necrosome was formed and low level of kinase activity was detected in the NP-40 soluble S25 fraction, TNF did not further increase this kinase activity (Figure 8C, top panel, lanes 1-4). The observed kinase activity was partly attributed to RIP1, as it was partially inhibited by Nec-1 (Figure 8D, compare lanes 3-4). In addition to kinase activity, we observed that RIP1-associated ubiquitination was strongly induced in the NP-40 insoluble compartments (Figure 8E). Paradoxically, the increase in RIP1-associated ubiquitination correlated with the recruitment of CYLD to RIP1 (Figure 8C, bottom panels). Inducible ubiquitination was not detected in the soluble fractions. Collectively, these results strongly suggest that RIP1 polyubiquitination within the necrosome critically regulates RIP1 kinase activation.

**Figure 9:**
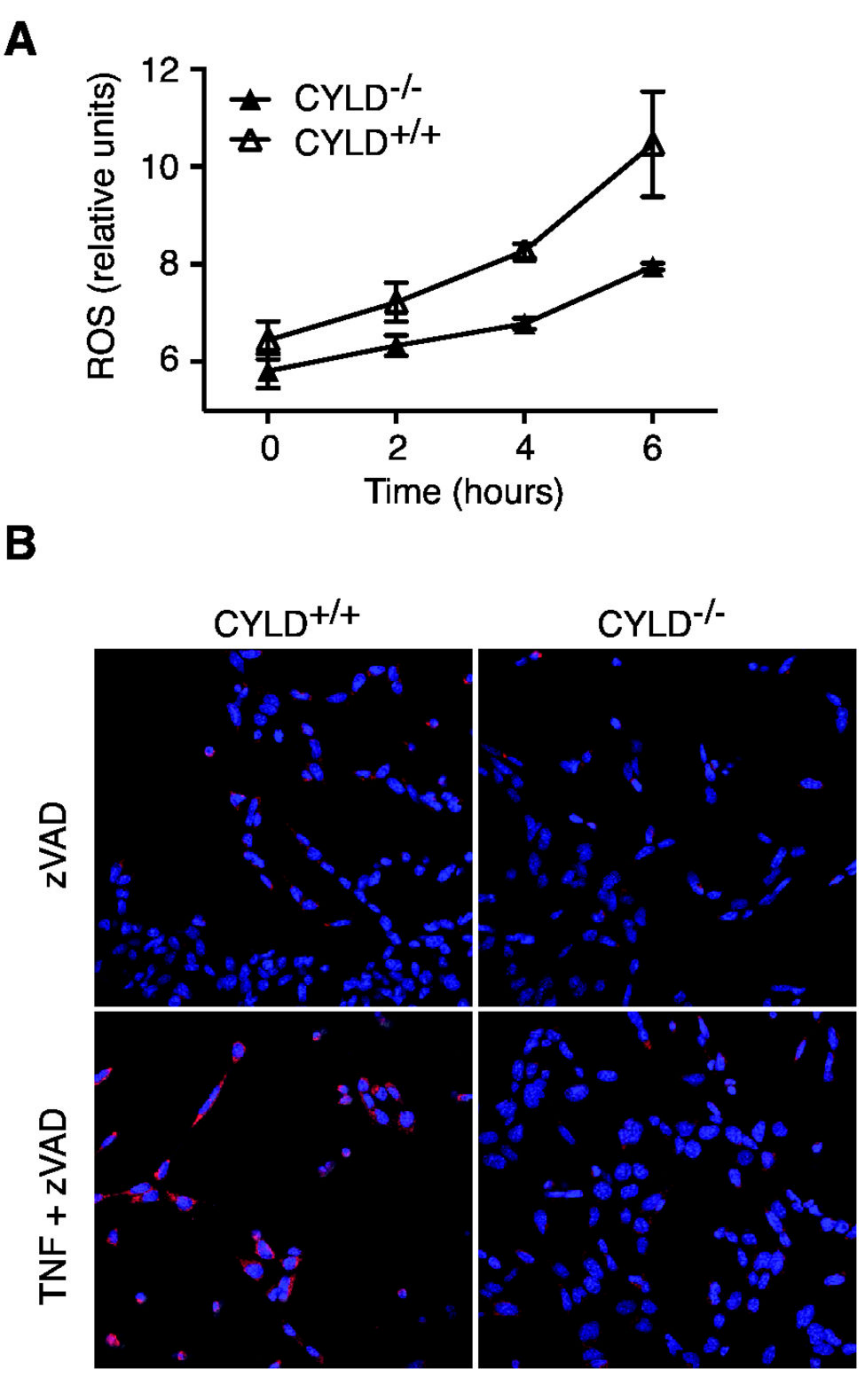
ROS production is reduced in CYLD^-/-^ MEFs. (**A**) CYLD^-/-^ or CYLD^+/+^ MEFs were stimulated with TNF and zVAD for the indicated times. ROS generation was determined by staining with CM-H _2_DCFDA as per manufacturer’s instructions. Results shown are average +/- SEM of triplicates. (**B**) CYLD^-/-^ or CYLD^+/+^ MEFs were stimulated with TNF and zVAD. ROS production was determined by staining with MitoSox as per manufacturer’s instructions.

### CYLD also controls downstream ROS production

The fact that the RIP1-RIP3 necrosome could form with only delayed kinetics and yet cell death was reduced in CYLD^-/-^ MEFs suggests that CYLD may also control a downstream step in programmed necrosis. Reactive oxygen species (ROS) production is a hallmark of necrosis. Indeed, we found that ROS production as measured by flow cytometry or by confocal microscopy was reduced in CYLD^-/-^ MEFs (Figure 9A-B). Therefore, despite relatively normal assembly of the necrosome, CYLD^-/-^ cells were defective for downstream signaling such as ROS production.

**Figure 10:**
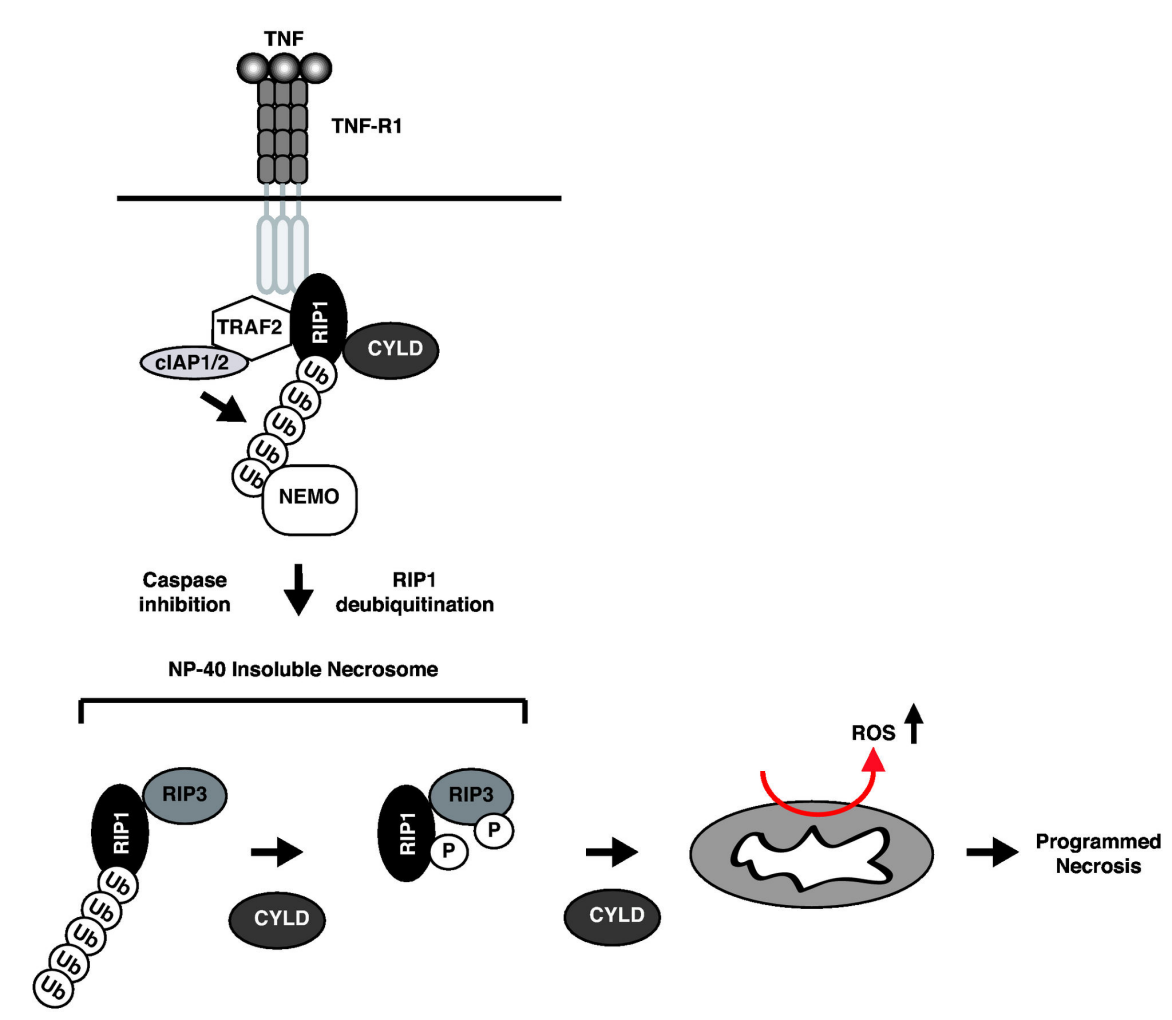
Schematic diagram of the proposed mechanism of CYLD-mediated necrosis. Although CYLD is recruited to the TNFR-1 complex, it dose not deubiquitinate RIP1 within the this compartment. Upon transition to the cytosol, CYLD deubiquitinates RIP1 within the NP-40 insoluble fraction to actively promote necrosome phosphorylation and activation. In addition to regulating RIP1 ubiquitination in the necrosome, CYLD also controls downstream events of necrosis such as ROS production.

## Discussion

Protein ubiquitination plays key roles in cell death and innate immune signaling pathways [46]. Polyubiquitinated RIP1 at the TNFR-1 complex was shown to sterically hinder binding of caspase 8 to inhibit apoptosis [20]. A similar role for polyubiquitinated RIP1 has been proposed to inhibit necrosome formation [24,30]. Hence, CYLD was thought to promote necrosis by de-ubiquitination of RIP1 at the TNFR-1 membrane complex. In contrast to this prevailing model, we show that although CYLD was recruited to the TNFR-1 complex, RIP1 ubiquitination at the TNFR-1 complex was unaffected in CYLD^-/-^ cells. Moreover, the RIP1-RIP3 necrosome was formed, albeit with delayed kinetics, in CYLD^-/-^ or CYLD knock-down cells. CYLD was recruited to the necrosome in a TNF-dependent manner. However, this interaction is likely to be indirect, as expression of CYLD in 293T cells or in baculovirus did not reveal a strong interaction between CYLD and RIP1 (data not shown). Adaptors such as the recently reported CLIPR-59 may mediate the interaction between RIP1 and CYLD [47].

In contrast to RIP1 ubiquitination in the TNFR-1 complex, RIP1 ubiquitination in the necrosome was indeed increased in the absence of CYLD. In particular, the necrosome isolated from the NP-40 insoluble fraction showed strong induction of ubiquitination in response to TNF, suggesting that CYLD may instead regulate RIP1 ubiquitination in this compartment (Figure 10). This model is consistent with the recent discovery that caspase 8-mediated cleavage of CYLD limits TNF-induced programmed necrosis [24], since caspase 8 is present in the necrosome, but not the TNFR-1 complex. Because CYLD is not essential for necrosome assembly, but rather facilitates its activation, our results may also explain why genetic inactivation or siRNA silencing of CYLD was not as effective as inactivating RIP1 or RIP3 in blocking programmed necrosis in cells or in FADD^-/-^ or caspase 8^-/-^ mice [12,13].

A consequence of increased RIP1 ubiquitination in CYLD^-/-^ cells is impaired RIP1 and RIP3 phosphorylation and activation. The polyubiquitin chains on RIP1 may sterically prevent autophosphorylation of RIP1 or limit access of an upstream RIP1 activating kinase. Alternatively, it may sterically restrict RHIM-mediated amyloid complex assembly, which facilitates kinase activation [45]. Regardless of the mechanism, it is surprising that CYLD deficiency led to a substantial reduction in necrosis and yet necrosome assembly was only marginally affected. The precise reason for this difference is unknown at present. However, it is noteworthy that the levels of RIP1, RIP3 and CYLD in the NP-40 fractions decreased as programmed necrosis ensued (Figure 7). While some of the loss could be attributed to relocation of these factors to the NP-40 insoluble fractions, direct lysis with SDS buffer revealed that protein degradation might also contribute to their loss (Figure 6A). This necrosis-induced degradation of signaling adaptors was impaired in CYLD^-/-^ cells. Moreover, ROS production was also impaired in CYLD^-/-^ MEFs. These results suggest that CYLD may have dual functions in controlling RIP1 ubiquitination at the necrosome as well as a yet to be identified downstream event in the necrosis signaling pathway.

## References

[1] Moriwaki K, Chan FK (2013) RIP3: a molecular switch for necrosis and inflammation. Genes Dev 27: 1640-1649. doi:10.1101/gad.223321.113. PubMed: 23913919. 23913919 PMC3744722

[2] Challa S, Chan FK (2010) Going up in flames: necrotic cell injury and inflammatory diseases. Cell Mol Life Sci 67: 3241-3253. doi:10.1007/s00018-010-0413-8. PubMed: 20532807. 20532807 PMC3051829

[3] Lin Y, Devin A, Rodriguez Y, Liu ZG (1999) Cleavage of the death domain kinase RIP by caspase-8 prompts TNF-induced apoptosis. Genes Dev 13: 2514-2526. doi:10.1101/gad.13.19.2514. PubMed: 10521396. 10521396 PMC317073

[4] Feng S, Yang Y, Mei Y, Ma L, Zhu DE et al. (2007) Cleavage of RIP3 inactivates its caspase-independent apoptosis pathway by removal of kinase domain. Cell Signal 19: 2056-2067. doi:10.1016/j.cellsig.2007.05.016. PubMed: 17644308. 17644308

[5] Chan FK, Shisler J, Bixby JG, Felices M, Zheng L et al. (2003) A role for tumor necrosis factor receptor-2 and receptor-interacting protein in programmed necrosis and antiviral responses. J Biol Chem 278: 51613-51621. doi:10.1074/jbc.M305633200. PubMed: 14532286. 14532286

[6] Zhang DW, Shao J, Lin J, Zhang N, Lu BJ et al. (2009) RIP3, an Energy Metabolism Regulator that Switches TNF-Induced Cell Death from Apoptosis to Necrosis. Science, 325: 332–6. PubMed: 19498109. 19498109 10.1126/science.1172308

[7] Zhang H, Zhou X, McQuade T, Li J, Chan FK et al. (2011) Functional complementation between FADD and RIP1 in embryos and lymphocytes. Nature 471: 373-376. doi:10.1038/nature09878. PubMed: 21368761. 21368761 PMC3072026

[8] Oberst A, Dillon CP, Weinlich R, McCormick LL, Fitzgerald P et al. (2011) Catalytic activity of the caspase-8-FLIP(L) complex inhibits RIPK3-dependent necrosis. Nature 471: 363-367. doi:10.1038/nature09852. PubMed: 21368763. 21368763 PMC3077893

[9] Kaiser WJ, Upton JW, Long AB, Livingston-Rosanoff D, Daley-Bauer LP et al. (2011) RIP3 mediates the embryonic lethality of caspase-8-deficient mice. Nature 471: 368-372. doi:10.1038/nature09857. PubMed: 21368762. 21368762 PMC3060292

[10] Ch’en IL, Tsau JS, Molkentin JD, Komatsu M, Hedrick SM (2011) Mechanisms of necroptosis in T cells. J Exp Med. 10.1084/jem.20110251 PMC3135356 21402742

[11] Sun L, Wang H, Wang Z, He S, Chen S et al. (2012) Mixed lineage kinase domain-like protein mediates necrosis signaling downstream of RIP3 kinase. Cell 148: 213-227. doi:10.1016/j.cell.2011.11.031. PubMed: 22265413. 22265413

[12] Bonnet MC, Preukschat D, Welz PS, van Loo G, Ermolaeva MA et al. (2011) The adaptor protein FADD protects epidermal keratinocytes from necroptosis in vivo and prevents skin inflammation. Immunity 35: 572-582. doi:10.1016/j.immuni.2011.08.014. PubMed: 22000287. 22000287

[13] Welz PS, Wullaert A, Vlantis K, Kondylis V, Fernández-Majada V et al. (2011) FADD prevents RIP3-mediated epithelial cell necrosis and chronic intestinal inflammation. Nature 477: 330-334. doi:10.1038/nature10273. PubMed: 21804564. 21804564

[14] Vanlangenakker N, Vanden Berghe T, Bogaert P, Laukens B, Zobel K et al. (2011) cIAP1 and TAK1 protect cells from TNF-induced necrosis by preventing RIP1/RIP3-dependent reactive oxygen species production. Cell Death Differ 18: 656-665. doi:10.1038/cdd.2010.138. PubMed: 21052097. 21052097 PMC3131911

[15] Cho Y, McQuade T, Zhang HB, Zhang JK, Chan FKM (2011) RIP1-Dependent and Independent Effects of Necrostatin-1 in Necrosis and T Cell Activation. PLOS ONE 6: e23209 PubMed: 21853090. 21853090 10.1371/journal.pone.0023209 PMC3154273

[16] Cho YS, Challa S, Moquin D, Genga R, Ray TD et al. (2009) Phosphorylation-driven assembly of the RIP1-RIP3 complex regulates programmed necrosis and virus-induced inflammation. Cell 137: 1112-1123. doi:10.1016/j.cell.2009.05.037. PubMed: 19524513. 19524513 PMC2727676

[17] He S, Wang L, Miao L, Du F, Zhao L et al. (2009) Receptor Interacting Protein Kinase-3 Determines Cellular Necrotic Response to TNF-alpha. Cell 137: 1100-1111. doi:10.1016/j.cell.2009.05.021. PubMed: 19524512. 19524512

[18] Zhao J, Jitkaew S, Cai Z, Choksi S, Li Q et al. (2012) Mixed lineage kinase domain-like is a key receptor interacting protein 3 downstream component of TNF-induced necrosis. Proc Natl Acad Sci U S A 109: 5322-5327. doi:10.1073/pnas.1200012109. PubMed: 22421439. 22421439 PMC3325682

[19] Wang Z, Jiang H, Chen S, Du F, Wang X (2012) The mitochondrial phosphatase PGAM5 functions at the convergence point of multiple necrotic death pathways. Cell 148: 228-243. doi:10.1016/j.cell.2011.11.030. PubMed: 22265414. 22265414

[20] O’Donnell MA, Legarda-Addison D, Skountzos P, Yeh WC, Ting AT (2007) Ubiquitination of RIP1 regulates an NF-kappaB-independent cell-death switch in TNF signaling. Curr Biol 17: 418-424. doi:10.1016/j.cub.2007.01.027. PubMed: 17306544. 17306544 PMC1868513

[21] Varfolomeev E, Blankenship JW, Wayson SM, Fedorova AV, Kayagaki N et al. (2007) IAP antagonists induce autoubiquitination of c-IAPs, NF-kappaB activation, and TNFalpha-dependent apoptosis. Cell 131: 669-681. doi:10.1016/j.cell.2007.10.030. PubMed: 18022362. 18022362

[22] Vince JE, Wong WW, Khan N, Feltham R, Chau D et al. (2007) IAP antagonists target cIAP1 to induce TNFalpha-dependent apoptosis. Cell 131: 682-693. doi:10.1016/j.cell.2007.10.037. PubMed: 18022363. 18022363

[23] Bertrand MJ, Milutinovic S, Dickson KM, Ho WC, Boudreault A et al. (2008) cIAP1 and cIAP2 facilitate cancer cell survival by functioning as E3 ligases that promote RIP1 ubiquitination. Mol Cell 30: 689-700. doi:10.1016/j.molcel.2008.05.014. PubMed: 18570872. 18570872

[24] O’Donnell MA, Hase H, Legarda D, Ting AT (2012) NEMO inhibits programmed necrosis in an NFkappaB-independent manner by restraining RIP1. PLOS ONE 7: e41238. doi:10.1371/journal.pone.0041238. PubMed: 22848449. 22848449 PMC3406058

[25] Massoumi R (2010) Ubiquitin chain cleavage: CYLD at work. Trends Biochem Sci 35: 392-399. doi:10.1016/j.tibs.2010.02.007. PubMed: 20347313. 20347313

[26] Hitomi J, Christofferson DE, Ng A, Yao J, Degterev A et al. (2008) Identification of a molecular signaling network that regulates a cellular necrotic cell death pathway. Cell 135: 1311-1323. doi:10.1016/j.cell.2008.10.044. PubMed: 19109899. 19109899 PMC2621059

[27] Lu JV, Walsh CM (2012) Programmed necrosis and autophagy in immune function. Immunol Rev 249: 205-217. doi:10.1111/j.1600-065X.2012.01147.x. PubMed: 22889224. 22889224 PMC4030387

[28] Han J, Zhong CQ, Zhang DW (2011) Programmed necrosis: backup to and competitor with apoptosis in the immune system. Nat Immunol 12: 1143-1149. doi:10.1038/ni.2159. PubMed: 22089220. 22089220

[29] Vandenabeele P, Galluzzi L, Vanden Berghe T, Kroemer G (2010) Molecular mechanisms of necroptosis: an ordered cellular explosion. Nat Rev Mol Cell Biol 11: 700-714. doi:10.1038/nrm2970. PubMed: 20823910. 20823910

[30] O’Donnell MA, Ting AT (2011) RIP1 comes back to life as a cell death regulator in TNFR1 signaling. FEBS J 278: 877-887. doi:10.1111/j.1742-4658.2011.08016.x. PubMed: 21232018. 21232018 PMC3051001

[31] O’Donnell MA, Perez-Jimenez E, Oberst A, Ng A, Massoumi R et al. (2011) Caspase 8 inhibits programmed necrosis by processing CYLD. Nat Cell Biol 13: 1437-1442. doi:10.1038/ncb2362. PubMed: 22037414. 22037414 PMC3229661

[32] Harper N, Hughes M, MacFarlane M, Cohen GM (2003) Fas-associated death domain protein and caspase-8 are not recruited to the tumor necrosis factor receptor 1 signaling complex during tumor necrosis factor-induced apoptosis. J Biol Chem 278: 25534-25541. doi:10.1074/jbc.M303399200. PubMed: 12721308. 12721308

[33] Micheau O, Tschopp J (2003) Induction of TNF receptor I-mediated apoptosis via two sequential signaling complexes. Cell 114: 181-190. doi:10.1016/S0092-8674(03)00521-X. PubMed: 12887920. 12887920

[34] Wang L, Du F, Wang X (2008) TNF-alpha induces two distinct caspase-8 activation pathways. Cell 133: 693-703. doi:10.1016/j.cell.2008.03.036. PubMed: 18485876. 18485876

[35] Reiley WW, Zhang M, Jin W, Losiewicz M, Donohue KB et al. (2006) Regulation of T cell development by the deubiquitinating enzyme CYLD. Nat Immunol 7: 411-417. doi:10.1038/ni1315. PubMed: 16501569. 16501569

[36] Chan FK (2012) Fueling the flames: Mammalian programmed necrosis in inflammatory diseases. Cold Spring Harb Perspect Biol 4: ([MedlinePgn:]) PubMed: 23125016. 10.1101/cshperspect.a008805 PMC3536335 23125016

[37] Wright A, Reiley WW, Chang M, Jin W, Lee AJ et al. (2007) Regulation of early wave of germ cell apoptosis and spermatogenesis by deubiquitinating enzyme CYLD. Dev Cell 13: 705-716. doi:10.1016/j.devcel.2007.09.007. PubMed: 17981138. 17981138

[38] Wertz IE, O’Rourke KM, Zhou H, Eby M, Aravind L et al. (2004) De-ubiquitination and ubiquitin ligase domains of A20 downregulate NF-kappaB signalling. Nature 430: 694-699. doi:10.1038/nature02794. PubMed: 15258597. 15258597

[39] Shembade N, Ma A, Harhaj EW (2010) Inhibition of NF-kappaB Signaling by A20 Through Disruption of Ubiquitin Enzyme Complexes. Science 327: 1135-1139. doi:10.1126/science.1182364. PubMed: 20185725. 20185725 PMC3025292

[40] Green DR, Oberst A, Dillon CP, Weinlich R, Salvesen GS (2011) RIPK-dependent necrosis and its regulation by caspases: a mystery in five acts. Mol Cell 44: 9-16. doi:10.1016/j.molcel.2011.09.003. PubMed: 21981915. 21981915 PMC3192321

[41] Vanlangenakker N, Bertrand MJ, Bogaert P, Vandenabeele P, Vanden Berghe T (2011) TNF-induced necroptosis in L929 cells is tightly regulated by multiple TNFR1 complex I and II members. Cell Death. Drosophila Inf Serv 2: e230. 10.1038/cddis.2011.111 PMC3223695 22089168

[42] Moujalled DM, Cook WD, Okamoto T, Murphy J, Lawlor KE et al. (2013) TNF can activate RIPK3 and cause programmed necrosis in the absence of RIPK1. Cell Death. Drosophila Inf Serv 4: e465. 10.1038/cddis.2012.201 PMC3563989 23328672

[43] Ea CK, Deng L, Xia ZP, Pineda G, Chen ZJ (2006) Activation of IKK by TNFalpha requires site-specific ubiquitination of RIP1 and polyubiquitin binding by NEMO. Mol Cell 22: 245-257. doi:10.1016/j.molcel.2006.03.026. PubMed: 16603398. 16603398

[44] Li H, Kobayashi M, Blonska M, You Y, Lin X (2006) Ubiquitination of RIP is required for tumor necrosis factor alpha-induced NF-kappaB activation. J Biol Chem 281: 13636-13643. doi:10.1074/jbc.M600620200. PubMed: 16543241. 16543241

[45] Li J, McQuade T, Siemer AB, Napetschnig J, Moriwaki K et al. (2012) The RIP1/RIP3 necrosome forms a functional amyloid signaling complex required for programmed necrosis. Cell 150: 339-350. doi:10.1016/j.cell.2012.06.019. PubMed: 22817896. 22817896 PMC3664196

[46] Harhaj EW, Dixit VM (2012) Regulation of NF-kappaB by deubiquitinases. Immunol Rev 246: 107-124. doi:10.1111/j.1600-065X.2012.01100.x. PubMed: 22435550. 22435550 PMC3540820

[47] Fujikura D, Ito M, Chiba S, Harada T, Perez F et al. (2012) CLIPR-59 regulates TNF-alpha-induced apoptosis by controlling ubiquitination of RIP1. Cell Death. Drosophila Inf Serv 3: e264. 10.1038/cddis.2012.3 PMC3288345 22297296

[48] Challa S, Woelfel M, Guildford M, Moquin D, Chan FK (2010) Viral cell death inhibitor MC159 enhances innate immunity against vaccinia virus infection. J Virol 84: 10467-10476. doi:10.1128/JVI.00983-10. PubMed: 20702623. 20702623 PMC2950582

